# A Novel Murine Model for the *In Vivo* Study of Transdermal Drug Penetration

**DOI:** 10.1100/2012/543536

**Published:** 2012-01-04

**Authors:** Gábor Erős, Petra Hartmann, Szilvia Berkó, Eszter Csizmazia, Erzsébet Csányi, Anita Sztojkov-Ivanov, István Németh, Piroska Szabó-Révész, István Zupkó, Lajos Kemény

**Affiliations:** ^1^Department of Dermatology and Allergology, University of Szeged, 6720 Szeged, Hungary; ^2^Institute of Surgical Research, University of Szeged, 6720 Szeged, Hungary; ^3^Department of Pharmaceutical Technology, University of Szeged, 6720 Szeged, Hungary; ^4^Department of Pharmacodynamics and Biopharmacy, University of Szeged, 6720 Szeged, Hungary; ^5^Dermatological Research Group of the Hungarian Academy of Sciences and the University of Szeged, 6720 Szeged, Hungary

## Abstract

Enhancement of the transdermal penetration of different active agents is an important research goal. Our aim was to establish a novel *in vivo* experimental model which provides a possibility for exact measurement of the quantity of penetrated drug. The experiments were performed on SKH-1 hairless mice. A skin fold in the dorsal region was fixed with two fenestrated titanium plates. A circular wound was made on one side of the skin fold. A metal cylinder with phosphate buffer was fixed into the window of the titanium plate. The concentration of penetrated drug was measured in the buffer. The skin fold was morphologically intact and had a healthy microcirculation. The drug appeared in the acceptor buffer after 30 min, and its concentration exhibited a continuous increase. The presence of ibuprofen was also detected in the plasma. In conclusion, this model allows an exact *in vivo* study of drug penetration and absorption.

## 1. Introduction

The ability of various agents to undergo transdermal penetration is a crucial question in pharmaceutics. Transdermal drug delivery is a useful alternative pathway for therapeutic agents which are prone to decompose in the gastrointestinal tract and permits the achievement of relatively high local drug concentrations without systemic side effects. However, transdermal drug delivery *per se* is possible for few drugs [1]. Accordingly, considerable research efforts are focused on physical [2] or chemical [3] methods with the aim of the enhancement of transdermal penetration. Studies of drug permeation and of different penetration-facilitating methods require appropriate models. The Franz diffusion cell [4] is an accepted and widely applied model for dermal and transdermal delivery. Reconstructed human epidermis, which contains viable cells, is also available and suitable for permeation studies [5]. Other types of *in vitro* models include isolated and perfused porcine forelimbs [6, 7]. Although *in vitro* tests are frequently used and approved methods and their application is strongly encouraged in order to reduce the need for both animal and human studies, at the present stage, *in vivo* examinations cannot be completely dispensed within pharmacological and dermatological studies. Many different types of *in vivo *methods have been developed. Some of them are based on the monitoring of the effects of penetrating agents. In these models, pathological conditions are induced, for example, renovascular hypertension [8] or pain [9], and amelioration of the disorder is considered proof of effective penetration. In other models, the presence of the drug in the skin is revealed or its impact on the structure or on certain functions of the skin is demonstrated. In such experiments, samples are taken from the stratum corneum with a tape stripping technique, and the penetration is analysed via different types of spectroscopy [10, 11]. Other methods, such as the assessment of transepidermal water loss, laser scanning microscopy, and laser Doppler flowmetry, can also be utilized *in vivo *in order to determine various changes in the skin [10, 12]. Nevertheless, in contrast with the Franz diffusion cell, these techniques do not provide a possibility for exact quantitative measurement of the extent of penetration. Accordingly, there appears to be a need for broadening of the toolbox of models available for permeation studies. 

Our objective was to develop a novel experimental model which permits precise determination of the quantity of drug penetrating living full-thickness skin with a functioning microcirculation, thereby allowing the acquisition of information on the absorption of the studied agent. Moreover, the detection of local side effects, skin irritation, or any other type of alteration in the skin is possible.

We chose the dorsal skin fold chamber model, which has been used in a considerable number of experimental physiological and pathophysiological studies for more than twenty years [13]. A modified version of this experimental setup seemed to provide an effective means of performing *in vivo* examinations of permeation. A topical ibuprofen-containing formulation was applied to test the model. This agent is a potent non steroidal anti-inflammatory drug which is stable in solution and, therefore, can be used in topical gel form and which has already been utilized in several penetration studies [14, 15]. Beside the assessment of the penetration and absorption of the drug, we planned to study the morphology and the microcirculatory characteristics of the skin in order to confirm the feasibility of the model and to estimate the potential area of its application.

## 2. Materials and Methods

### 2.1. Materials

Ibuprofen was from Sigma (St. Louis, Mo, USA), Carbopol 971 from BF Goodrich Co., USA, and polyethylene glycol 400 and triethanolamine from Hungaropharma Ltd. (Hungary). Methanol was of HPLC-gradient grade (Scharlau Chemie S.A., Barcelona, Spain), glacial acetic acid and ammonium hydroxide (Merck, Darmstadt, Germany) and potassium dihydrogenphosphate (Molar Chemicals KFT., Budapest, Hungary) were of analytical grade, and orthophosphoric acid and naproxen (Fluka, Sigma-Aldrich) were of HPLC grade. Deionized HPLC-grade water was generated by using a Milli-Q System from Millipore (Bedford, Mass, USA).

### 2.2. Preparation of Ibuprofen Gel

**Figure 1:**
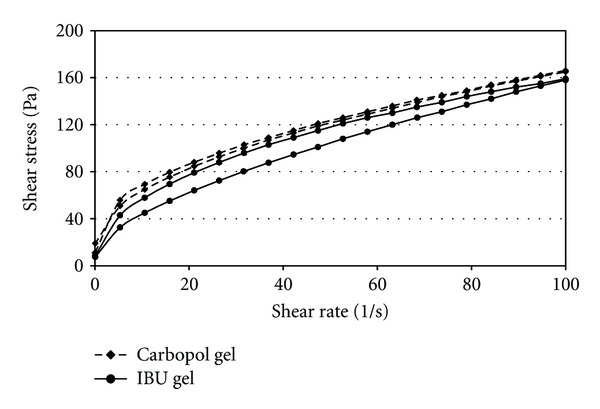
The flow curves of the Carbopol 971 hydrogel (control preparation) and ibuprofen containing formulation.

The formulation containing 5% w/w ibuprofen was prepared by the following procedures. A 3% w/w Carbopol 971 hydrogel was prepared, and ibuprofen dissolved in polyethylene glycol 400 with intensive mixing was added to this gel. The pH was adjusted with the use of triethanolamine. The control preparation had the same composition, but without ibuprofen. Prior to *in vivo *application, the preparation was carefully examined. The gel was transparent the pH was found to be 6.2. The rheological profile of the samples was studied by Paar Physica MCR101 rheometer (Anton Paar GmbH, Austria). A cone-plate measuring device was used in which the cone angle was 1 degree and the thickness of the sample was 0.046 mm in the middle of the cone. The measurements were carried out at 25°C. Flow curves of the different samples were determined. The shear rate was increased from 0.1 to 100 1/s (up curve) and then decreased from 100 to 0.1 1/s (down curve). The shearing time was 300 s in case of both segments. Rheology is the study of how matters deform and flow under the influence of external forces. This deformation is strongly influenced by the inner structure; in this way rheological investigations are useful tools to describe different materials. In case of flow curves, shear stress was measured as the function of increasing and decreasing shear rate.  Figure 1 demonstrates the flow curves of ibuprofen-containing sample (IBU gel) and ibuprofen-free Carbopol 971 hydrogel which served as control preparation. According to our measurements, incorporation of active pharmaceutical ingredient (ibuprofen) did not influence the viscosity of the formulation remarkably.

### 2.3. Animals

The experiments were performed on 15-week-old male SKH-1 hairless mice (body weight (bw): 36–42 g). The animals were housed in plastic cages in a thermoneutral environment with a 12 h light-dark cycle and had access to standard laboratory chow and water *ad libitum*. All interventions were in full accordance with the NIH guidelines. The procedures and protocols applied were approved by the Ethical Committee for the Protection of Animals in Scientific Research at the University of Szeged (Permit number: I-74-9/2011.MÁB).

### 2.4. Implantation of the Dorsal Skin Fold Chamber

**Figure 2:**
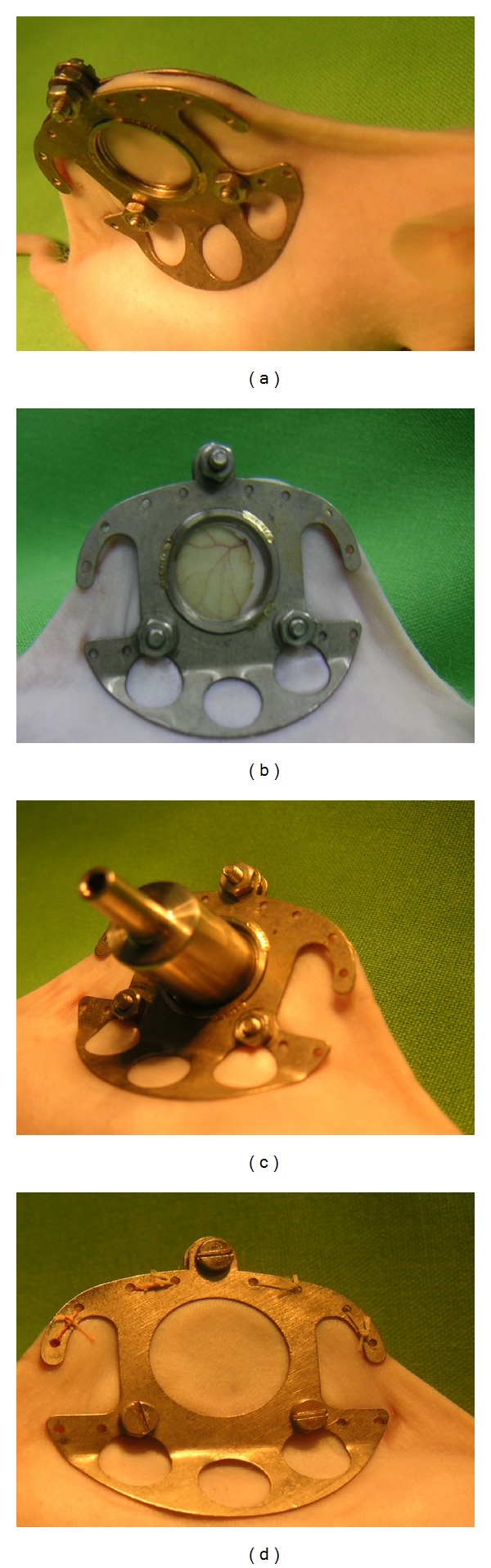
Steps of application of skin fold chamber. (a) Titanium frames on both sides of the skin fold; (b) circular, full-dermal-thickness wound; (c) metal cylinder containing the acceptor buffer (wounded side); (d) nonwounded side.

Prior to the surgical intervention, the animals were examined. Mice with any type of injury or dermatological disorder in the dorsal region were discarded. The animals were anaesthetized intraperitoneally with a mixture of ketamine (90 mg/kg bw) and xylazine (25 mg/kg bw). Two holding stitches were inserted in the dorsal midline and exerted moderate tension so as to form a skin fold. The entire process of the operation was performed under sterile circumstances. Two symmetrical titanium frames (IROLA GmbH, Schonach, Germany) were then applied to sandwich the extended double layer of the skin (Figure 2(a)). The skin fold was fixed to one of the metal frames with sutures and sandwiched securely between the frames by means of three nuts and bolts. A circular full-dermal-thickness wound was formed on one side of the skin fold by removal of the complete skin down to the musculus panniculus carnosus, thereby creating a wound area of 66–70 mm^2^ (Figure 2(b)). Microsurgical technique was used for the creation of the wound. The nonwounded skin on the opposite side still consisted of epidermis, dermis and striated skin muscle. The wounded side was covered with a removable thin plate of glass incorporated in one of the titanium frames. After the operation, the animals were returned to their cages. 24 h later, they were again examined. Signs of bleeding, infection, or any other type of disorder of the wound or the skin fold were considered exclusion criteria. The animals were anaesthetized, the glass plate was removed, and a stainless steel cylinder with a volume of 1 mL was fixed into the window of the titanium frame on the wounded side (Figure 2(c)). The wound edges precisely matched the aperture of the cylinder. (The creation of the wound was achieved after marking the area with a standardized circular ink stamp the diameter of which was the same that as of the cylinder). The opposite, nonwounded side (Figure 2(d)) served for the application of the study formulation. 

### 2.5. *In Vivo* Examination of the Penetration

The anaesthetized mice were placed in a lateral position on a heating pad for maintenance of the body temperature at 37°C. The nonwounded side was downwards. Small supplementary doses of ketamine and xylazine were administered when necessary. 0.1 g of the study formulation was applied to the nonwounded side. This side was then covered with a nonpermeable film. 1 mL of phosphate-buffered saline (PBS) (pH = 7.37) was added to the metal cylinder. The observation period lasted for 6 h. After 30 min, 1 h, 2 h, 3 h, 4 h, 5 h, and 6 h, the PBS was removed from the cylinder (a small piece of soft plastic tube was placed onto the tip of the sample-taking needle in order not to damage the tissue) and was replaced by fresh PBS. The samples were stored at −20°C, and the concentration of the penetrated drug was measured by means of high-performance liquid chromatography (HPLC). At the end of the observation period, the abdominal cavity was opened, and a blood sample was taken from the inferior cava vein with a needle and syringe containing 250 IU of Naheparin. The blood was then centrifuged at 3500 g for 5 min in order to separate the cellular components. The plasma was removed, stored at −20°C, and analyzed by HPLC. The tissue layer in the window of the skin fold chamber was removed and saved for histological examination. At the end of the experiment, the animals were euthanized with an overdose of ketamine.

### 2.6. Groups

The mice were randomly allocated into the following groups. Group 1 (*n* = 6) served for the study of microcirculation. A dorsal skin fold was created, and the animals were observed as described above, but no study formulation was applied to the skin. After 6 h of observation, the metal cylinder was removed and the microcirculation of the skin was monitored by means of intravital videomicroscopy. In group 2 (*n* = 8), a control gel without drug was applied to the skin. The animals in group 3 (*n* = 8) received a formulation containing ibuprofen. In these groups, tissue samples for histological analysis were also taken. In group 4 (*n* = 8) and group 5 (*n* = 8), a gel containing ibuprofen was applied, and the animals were sacrificed after 1 h or 3 h, respectively, in order to obtain blood samples at these times.

### 2.7. Intravital Videomicroscopy

The microcirculation was visualized with a fluorescence intravital videomicroscope equipped with a 100 W mercury lamp (Axiotech vario, Zeiss, Jena, Germany). The anaesthetized mice received a retrobulbar injection of 80 *μ*L 2% fluorescein isothiocyanate-labeled dextran (molecular weight 150 kD; Sigma Chemicals, USA). After this injection, a blue (450–490 nm) filter set allowed analysis of the microcirculation by the epi-illumination technique, using an Achroplan 20x water immersion objective. The objective was directed towards both the wounded and nonwounded sides of the skin fold. During examinations, the tissue was superfused with 37°C saline. The intravital microscopic images were recorded with a charge-coupled device videocamera (AVT-BC 12, AVT Horn, Aalen, Germany) attached to an S-VHS video recorder (Panasonic AG-MD830) and a personal computer. Quantitative assessment of the microcirculatory parameters was performed offline with frame-to-frame analysis, using image analysis software (IVM, Pictron Ltd., Budapest, Hungary). The red blood cell velocity (RBCV, *μ*m/s) was measured in 5 separate fields of view, in at least 6 capillaries. The perfusion rate (PR) was also monitored. The lengths of the perfused, functioning vessels were determined and referred to the entire vessel length measured in the field of view. The PR was given as a percentage and was determined in at least 3 different fields of view in each animal.

### 2.8. Histology

The tissue layer in the window of the titanium chamber on the nonwounded side was excised. The biopsies were fixed in a 4% buffered solution of formaldehyde and embedded in paraffin. The routine haematoxylin-eosin-stained 4 *μ*m thick sections were subjected to histological examination with a Zeiss Axioscope Z1. AxioVision software (Zeiss Corp.) was used for the measurement of epidermal thickness. The evaluation was performed in coded sections by a professional pathologist (I. Németh).

### 2.9. HPLC

The measurements were performed under the following chromatographic conditions. The HPLC instrumentation included a Shimadzu CBM-20A/20Alite system controller, a Shimadzu LC-20AD solvent delivery system, a Shimadzu DGU-20A3 on-line degasser, a Shimadzu SPD-M20A UV/VIS photodiode array detector, and a Shimadzu CTO-20A column oven. The chromatographic system was equipped with a Rheodyne Model 7725i injector (Cotati, Calif, USA) with a 20 *μ*L loop. The chromatographic data were collected and processed by using the Shimadzu LCsolution software.

Chromatographic separations were performed on a Hichrom Kromasil 100-5C8 (150 mm × 4.6 mm, 5 *μ*m particle size) reversed-phase column (Berkshire, UK), preceded by a Hichrom Kromasil 100-5C8 guard column. The mobile phase consisted of 0.025 M KH_2_PO_4_ buffer (pH = 2.7)-methanol. In the gradient elution of ibuprofen, the methanol content of the mobile phase was increased linearly from 40 to 80% during the first 5 min and maintained at that level for a further 8 min. After the 13-min run, the methanol content was returned to 40% during 2 min, and the column was re-equilibrated for at least 5 min before the next injection. A constant flow rate of 1.00 mL/min, a column temperature of 40°C, and a sample volume of 20 *μ*L were used for all injections. The UV detector was set at 215 nm. The mobile phase was degassed by ultrasonication before use. The samples were prepared as follows. An aliquot of plasma (200 *μ*L) was combined with 20 *μ*L 0.02 ng/mL naproxen as internal standard working solution. The sample was acidified by the addition of 80 *μ*L 2% H_3_PO_4_ and diluted with 200 *μ*L 5% acetic acid solution. The mixture was centrifuged at 12000 rpm for 10 min at 4°C, and the supernatant was loaded onto the Superclean LC-Ph SPE tube (Sigma-Aldrich), which had previously been conditioned with 3 mL methanol and 3 mL 5% acetic acid solution, under a vacuum extraction manifold device. The cartridge was washed with 3 mL H_2_O and 2 mL 5% acetic acid solution: MeOH (95 : 5 v/v). The ibuprofen retained in the cartridge, was eluted with 2 mL 5% NH_4_OH solution: MeOH (5 : 95 v/v), and then 1 mL MeOH into a glass test-tube. The eluate was evaporated to dryness under a stream of nitrogen at 40°C and reconstituted with 200 *μ*L water: methanol (50 : 50 v/v), and 20 *μ*L was injected into the HPLC system. PBS calibration standard solutions was prepared by appropriate dilution of ibuprofen working solutions with PBS in the concentration range 0.0001–0.02 ng/mL. Aliquots of 20 *μ*L PBS calibration standards and unknown PBS samples were injected directly into the HPLC system for analysis.

### 2.10. Statistical Analysis

Data analysis was performed with a statistical software package (SigmaStat for Windows, Jandel Scientific, Erkrath, Germany). The *t*-test was chosen for the analysis of differences between the microcirculatory data relating to the wounded and the nonwounded sides. Differences between the measurements at different times were determined by repeated measure ANOVA and the Bonferroni test as post hoc test. *P* < 0.05 was considered significant. The data are reported as mean values ± standard deviation (SD).

## 3. Results

### 3.1. Histological Analysis of the Skin Fold

**Figure 3:**
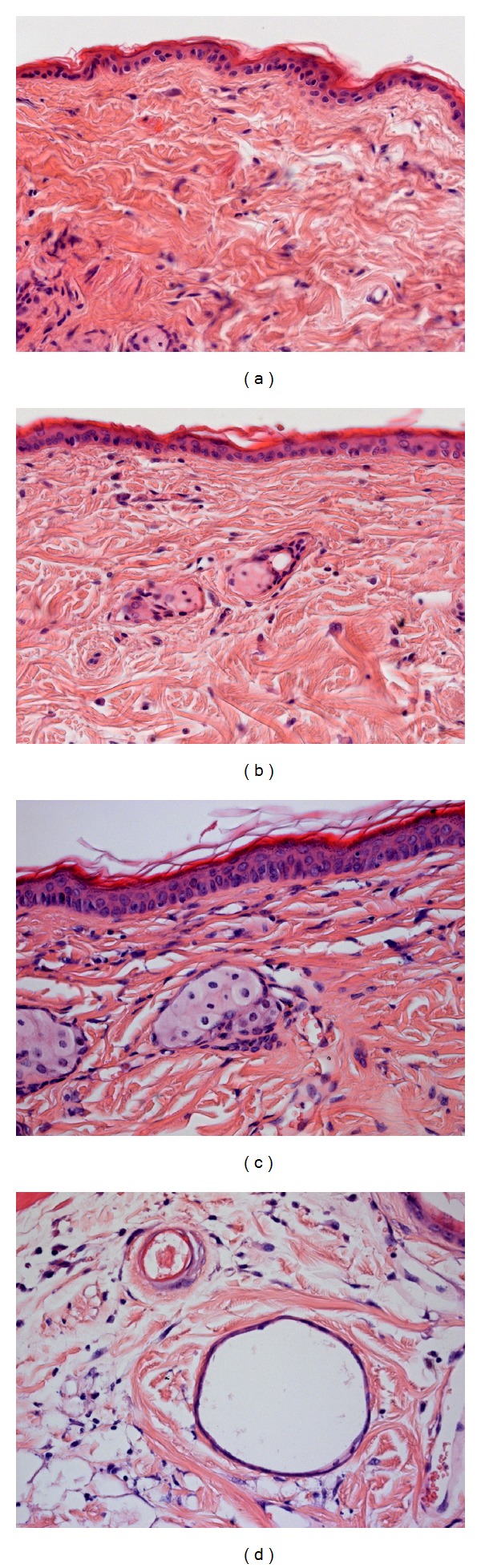
Photomicrographs of the skin from groups 2, 5, and 1, respectively, (H&E staining). (a) Group 2, treated with ibuprofen-free gel. (b) Group 5, exposed to ibuprofen-containing gel. (c) Group 1; no treatment was applied. (d) Atrophic folliculus.

The light microscopic evaluation demonstrated that the skin was healthy in all the studied groups. The photomicrographs reveal that the epidermis was preserved. Furthermore, no alterations were found in the dermis or subcutis (Figures 3(a), 3(b), and 3(c)). The folliculi were atrophic and occasionally cystic, proving the hairless status of the skin (Figure 3(d)). As concerns the epidermal thickness, no significant difference was found between the groups (data not shown).

### 3.2. Microcirculatory Parameters

The microcirculation was examined on both the nonwounded and wounded sides of the skin fold. In the former location, the vessels of the dermis close to the dermoepidermal line were visualized, while in the latter position the microcirculation of the striated muscle layer under the dermis was studied.  Table 1 demonstrates the RBCV, the mean values of which were found to be 502.48 and 595.41 *μ*m/s on the nonwounded and wounded sides, respectively. The statistical analysis did not reveal a mathematically significant difference between the sites. However, the data lay in a broad range, with minimum and maximum values of 178.0 and 957.0 and of 225.0 and 1061.0 *μ*m/s on the nonwounded and wounded sides, respectively.

The other studied parameter was the PR. High PR rates were measured on both sides (nonwounded: mean = 91.41, SD = 2.24; wounded: mean = 91.34, SD = 4.08), with no significant difference between the sides (Table 1).

### 3.3. Penetration and Absorption of Ibuprofen

**Figure 4:**
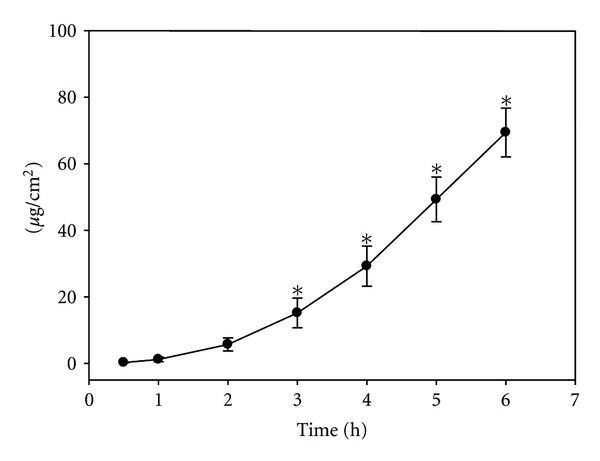
The penetration of ibuprofen. The curve displays mean values ± SD. The values are given in *μ*g/cm^2^. *P < 0.05 versus 30 min values.

The drug concentration of the buffer samples was referred to the area of the skin exposed to the study formulation.  Figure 4 illustrates the penetration of ibuprofen as a function of time. The penetration attained a measurable value by 30 min (mean = 0.244 *μ*g/cm^2^, SD = 0.13). The quantity of penetrated ibuprofen displayed a considerable elevation during the observation period. From 3 h on, the extent of penetration was significantly higher than that measured after 30 min. 2.13% of the applied active agent entered the acceptor phase by 6 h. The calculated flux at the end of the observation period was 11.57 *μ*g/cm^2^/h.

**Figure 5:**
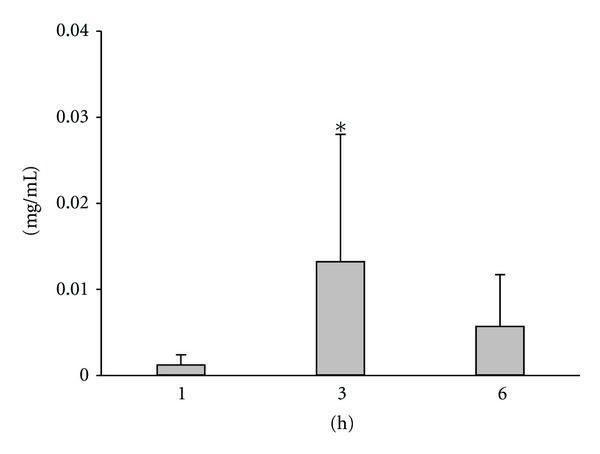
The absorption of ibuprofen. The bars display mean values ± SD. The values are given in mg/mL. *P < 0.05 versus 1 h values.

The ibuprofen concentration of the plasma was determined after 1, 3 and 6 h. The results are presented in Figure 5. The plasma concentration was relatively low after 1 h (mean = 0.00122 mg/mL, SD = 0.00102) but exhibited a significant increase after 3 h (mean = 0.0132, SD = 0.0128). However, a considerable decrease was found in the plasma concentration of the drug by 6 h (mean = 0.00571, SD = 0.0059).

## 4. Discussion

Although many different models are used for drug penetration studies, the choice of the most appropriate model is of great importance. Human skin would undoubtedly be the most predictive model for such investigations, but ethical problems and limited availability hamper its use. Hence, various animal models have been suggested as a substitute for human skin, though the drug penetration through animal skin may differ from that in human tissue [16]. It has been reported that percutaneous absorption in monkey [17] and in pig [18, 19] may closely predict that in human. However, such large animals are not readily available and are more expensive than smaller laboratory animals. Thus, rodent skin is widely applied. Due to the lack of fur, some authors have suggested the use of hairless rats [20], guinea pigs [21] and mice [22]. Hairlessness is an important point since it has been revealed that the number of folliculi, the opening diameter, and the follicular volume play major roles in drug penetration [23, 24]. Although it has been reported that murine skin is more permeable to certain drugs than human tissue [25], hairless mice have been applied in penetration studies both *in vitro *and *in vivo *[26, 27], and we, therefore, chose such animals for this study.

A further significant question is whether *in vitro* or *in vivo* methods should be applied to clarify a certain problem. Since the transdermal permeation of drugs and subsequent absorption or deep tissue delivery can be influenced by many different factors, *in vivo *models feature some advantages. *In vivo* examinations not only provide data on the uptake of the drug by the circulation and on the dermal metabolism of the active agent but also involve the use of skin the permeability of which differs from that of excised skin used *in vitro*. The elastic fibres which are cut during excision seem to constrict the follicular openings, thereby reducing the follicular reservoir and penetration pathway [28]. Our present study, therefore, aimed to develop an experimental model uniting the advantages of *in vitro *and *in vivo *methods, that is, the possibility of frequent sample taking in order to describe the penetration kinetics of the drug and the use of intact, living skin. A dorsal skin fold chamber is an accepted and sophisticated experimental model with which to study the microcirculation under different conditions, to test the biocompatibility of different materials [29], and to examine angiogenesis [30] and wound healing [31]. This chamber is tolerated by the animals even for weeks, and the literature findings and our own results demonstrate that application of this device does not impair the viability of the skin. The histological analysis did not reveal any sign of tissue damage in our experimental groups. Both the epidermis and the deeper layers appeared to be healthy. Thus, there is good reason to presume that the skin involved in this study had an intact barrier function and functioning cellular components. Beside morphological soundness, intravital microscopy confirmed a normal microcirculation in the applied skin fold. We examined both the wounded and the nonwounded sides of the skin fold. On the nonwounded side, the vessels directly under the epidermal layer can be monitored, while assessment of the wounded side visualizes the microcirculation of the “panniculus carnosus,” the striated skin muscle under the dermal layer. The perfusion rates of over 90% clearly showed that both sides had an adequate blood supply. No significant difference in RBCV was measured between the examined layers. However, the microcirculation displayed heterogeneity on both sides: vessels with low and high RBCV were seen in the same field of view. It has been described that timewise and spatial heterogeneity is characteristic of the microcirculation under compromised flow conditions [32]. Nevertheless, this phenomenon cannot be considered definitely pathologic, since laser Doppler's measurements have revealed that the patterns of flux exhibit a marked variability even in healthy skin [33]. In summary, this healthy microcirculatory network seems to provide an appropriate environment for the absorption of the penetrated drug.

In our model, the formation of the circular wound made the double-layered skin fold one layered on the area of the cylinder with the acceptor phase. Thus, the drug placed onto the nonwounded side had to penetrate one layer of epidermis, dermis, and a thin layer of striated muscle prior to entering the acceptor buffer. In the event of penetration, the active agent can be found in the acceptor phase. Our results indicated that the detection of the penetration was successful: the drug appeared in the acceptor buffer at a measurable rate after 30 min the penetration rate increased during the observation period. By the 6 h, a flux of 11.57 *μ*g/cm^2^/h was measured. Earlier, *in vitro* studies detected a higher flux of ibuprofen, with values of 20–30 *μ*g/cm^2^/h or higher [14, 34]. Further, our recent study also revealed a considerably higher rate of permeation of ibuprofen through human epidermis [35]. The lower quantity of penetrated drug in the present study can be explained in terms of different factors. In this experimental setup, the drug which crosses the epidermis enters the dermis, where ibuprofen is exposed to enzymatic breakdown. This enzymatic degradation leads to a lower bioavailability of the drug [36]. Further, ibuprofen can be taken up by the circulation. Our results confirmed the presence of the active agent in the plasma. Only that proportion of the drug not eliminated by degradation or removal in the circulation can be delivered towards the deeper layer and the acceptor phase.

The significant elevation in the plasma level of the drug after 3 h was followed by a decrease by 6 h. This can be explained by the relatively short half-life of ibuprofen, 2.2 h [37]. The ibuprofen-containing gel was applied only once, at the beginning of the experiment. No further doses were given to enhance the penetration. Hence, the processes of drug elimination (i.e., the metabolism and excretion of ibuprofen) appear to come to outweigh the influx of the active agent, resulting in a lowering plasma concentration.

Characterization of the extent of penetration and absorption is essential in order to predict the *in vivo *behaviour of a formulation or to study the efficacy of a penetration-enhancing method. However, other characteristics of the drug should also be considered. Ibuprofen is a lipophilic drug which forms a reservoir in the stratum corneum [36]. The accumulation of ibuprofen in the skin comprises 1-2 *μ*g/mg tissue [38]. Thus, removal and homogenization of the skin in order to determine the quantity of accumulated drug may provide useful data during future studies with the described model.

Overall, it can be concluded that the present experimental setup is suitable for the examination of transdermal drug penetration under *in vivo *circumstances. The model provides a possibility for consecutive sample taking from the acceptor phase, whereby repeated measurements can be performed in the same animal in order to characterize the kinetics of penetration. This lowers the number of animals required for a certain study. Other experimental setups, which allow the examination of blood samples only, may require more animals since repeated taking of blood quantities sufficient for the applied HPLC measurements may result in hemodynamic imbalances in mice. A further advantage of this setup is that the application of a skin fold chamber technically facilitates intravital microscopy by making plane tissue surfaces in the titanium windows. Monitoring of the microcirculation may provide additional information on the penetrating drug: as an example, vasoactive agents may lead to alterations in vessel diameter or RBCV. Furthermore, the application of a green filter set after the administration of rhodamine-6G allows the investigator to visualize neutrophilic granulocytes and to assess the leukocyte-endothelial interactions. In this way, potential irritative or inflammation-evoking effects of formulations can be revealed.

On the other hand, the model has the disadvantage that a continuous presence of an investigator is required for the assessment of the animals and for the maintenance of anaesthesia. Moreover, the observation period with the described model was only 6 h. Although this may be extended by few more hours, it is not possible to perform examinations whose duration achieves that with the Franz diffusion cell.

In conclusion, we have described a new model for *in vivo *penetration studies. This experimental setup allows repeated measurements in the same animal, simultaneous studies of penetration and absorption, and examinations of the microcirculation of the skin via which the penetration occurs. This model may be a useful addition in the armamentarium of penetration studies.

## Figures and Tables

**Table 1:** The microcirculatory parameters of the wounded- and nonwounded sides. Red blood cell velocity (RBCV) and perfusion rate (PR) values are reported as mean and standard deviation (SD). The unit of RBCV is *μ*m/s, the unit of PR is percent.

		Wounded side	Nonwounded side
RBCV	Mean	595.4	502.5
	SD	266.7	204.9
PR	Mean	91.3	91.4
	SD	4.1	2.2
